# Glia Maturation Factor β as a Novel Independent Prognostic Biomarker and Potential Therapeutic Target of Kidney Renal Clear Cell Carcinoma

**DOI:** 10.3389/fonc.2022.880100

**Published:** 2022-07-04

**Authors:** Tong Zhu, Tianyu Wang, Zijun Feng, Furong Gao, Jieping Zhang, Caixia Jin, Haibin Tian, Jingying Xu, Hao Chen, Qingjian Ou, Juan Wang, Guotong Xu, Lixia Lu

**Affiliations:** ^1^ Department of Ophthalmology of Shanghai Tongji Hospital, Laboratory of Clinical Visual Science of Tongji Eye Institute, School of Medicine, Tongji University, Shanghai, China; ^2^ Department of Biochemistry and Molecular Biology, Tongji University School of Medicine, Shanghai, China; ^3^ Department of Ophthalmology of Shanghai Tenth People’s Hospital, Tongji University School of Medicine, Shanghai, China; ^4^ Department of Pharmacology, Tongji University School of Medicine, Shanghai, China; ^5^ Department of Human Genetics, Tongji University School of Medicine, Shanghai, China

**Keywords:** kidney renal clear cell carcinoma, Glia maturation factor-beta, biomarker, prognosis, immune infiltration

## Abstract

Kidney renal clear cell carcinoma (KIRC) has the highest mortality rate and potential for invasion among renal cancers. The diagnosis and treatment of KIRC are becoming challenging because of its diverse pathogenic mechanisms. Glia (GMFB) is a highly conserved growth and differentiation factor for glia cells and neurons, and it is closely associated with neurodegenerative diseases. However, its role in KIRC remains unknown. The present study integrated bioinformatics approaches with suitable meta-analyses to determine the position of GMFB in KIRC. There was a significant decrease in *Gmfb* expression in KIRC kidneys compared with normal controls. *Gmfb* expression was negatively associated with pathologic stage, T and M stages, and histologic grade. Univariate and multivariate analyses showed that elevated *Gmfb* expression was an independent factor for a favorable prognosis. Furthermore, the nomogram verified that *Gmfb* is a low-risk factor for KIRC. Knockdown of *Gmfb* in Caki-2 cells increased viability and decreased p21 and p27 levels. Overexpression of *Gmfb* inhibited Caki-2 cell proliferation, migration, and invasion and decreased mitochondrial membrane potential. Gene ontology and Kyoto Encyclopedia of Genes and Genomes pathway enrichment analyses considering *Gmfb* co-expressed differentially expressed genes (DEGs) showed that collecting duct acid secretion and mineral absorption ranked were the most important upregulated and downregulated DEGs, respectively. The upregulated hub genes for DEGs were mainly involved in nucleosome assembly, nucleosome organization, and chromatin assembly, and the downregulated hub genes were primarily associated with keratinization. The ratio of tumor-infiltrating immune cells in KIRC tissues was evaluated using CIBERSORTx. The results showed that the *Gmfb* expression was significantly positively correlated with macrophage M2 cells and mast resting cell infiltration levels and negatively correlated with T follicular helper, T regulatory, and B plasma cell infiltration levels. The former cell types were associated with a beneficial outcome, while the latter had a worse outcome in patients with KIRC. In summary, this study identified GMFB as a novel independent biomarker and therapeutic target for KIRC, and it provides a helpful and distinct individualized treatment strategy for KIRC with a combination of molecular targets and tumor microenvironment.

## Introduction

Kidney cancer is one of the ten most common types of cancer worldwide (1, 2). It is estimated that 300,000 people worldwide develop kidney cancer each year, and approximately half of them die from this disease (3). Kidney renal clear cell carcinoma (KIRC), which originates from the epithelial cells of the proximal convoluted tubule of the nephron, has become one of the most rapidly evolving areas of oncology, accounting for approximately 75% of kidney cancers (3–5). In addition to being the most common subtype, KIRC has the highest aggressiveness and metastasis and the highest mortality rate (4), as well as the highest immunity and vascular infiltration of cancer types (6, 7).

The molecular mechanisms involved in KIRC pathogenesis are diverse (8), including direct chromatin configuration alteration, vasculogenesis, and glucose metabolism (8, 9). Over the past few years, many studies have focused on the oncogenes of renal cell carcinoma, resulting in the discovery of a repetitive mutation in KIRC (10). However, oncogenes and suppressors such as RAS genes, TP53, Rb, CDKN2A, BRAF, PIK3CA, PTEN, EGFR, and ERBB2, which are highly mutated in other adult epithelial cancers, play only a minor role in KIRC (10). The diagnosis and treatment of KIRC are becoming a challenge for the oncology community. Currently, the most reliable prognostic predictors for KIRC are T, N, and M stages (11). However, in these indicators, survival rates may vary considerably, even among patients with the same set of illnesses (11, 12). Therefore, there is an urgent need to identify novel, reliable prognostic biomarkers and therapeutic targets for patients with KIRC.

Recently, bioinformatics techniques have become crucial for deciphering the molecular mechanisms underlying KIRC. A considerable number of KIRC-related high-throughput microarray datasets and sequencing datasets have been accumulated, resulting in groundbreaking changes in KIRC research. Multi-omics have identified some genes, such as CHAC1 (13), METTL14 (14), P4HB (15), and FOXM1 (16), as novel prognostic indicators for KIRC. However, the analysis of the tumor microenvironment (TME) linked to the specific targets of KIRC is lacking.

It is well known that heterogeneity, an essential feature of tumors, may contribute to the distinct responses of cancer patients to the same treatment. Single-cell sequencing technology allows for dissecting tumor heterogeneity at the molecular and cellular levels. The TME is increasingly considered a target for drug treatment strategies (17). Recently, Hu et al. revealed intratumoral heterogeneity in KIRC, resulting in different clinical outcomes in single-cell transcriptomes (18).

Glia maturation factor-beta (GMFB), a highly conserved brain-specific protein, is a 17-kDa brain protein recognized as a growth and differentiation factor acting on neurons and glia (19, 20). It is essential for brain growth and development, and it is widely expressed in mammalian neuronal, glia, and parenchymal cells (21). GMFB is phosphorylated by protein kinase A (PKA) (19). Furthermore, PKA-phosphorylated GMFB inhibits extracellular signal-regulated kinase (ERK) and enhances p38 (22). It has been shown that the upregulation of GMFB is closely related to neurodegenerative disorders such as Parkinson’s disease (23). Our previous work revealed that GMFB functions as a novel independent prognostic biomarker and therapeutic target for liver hepatocellular carcinoma (LIHC) (24). However, the role of GMFB in KIRC remains unclear.

The present study employed The Cancer Genome Atlas (TCGA) database and the CIBERSORTx tool (https://cibersortx.stanford.edu) to explore the potential effects of GMFB and GMFB-related TME, and the significant findings of molecular signatures were confirmed by quantitative real-time PCR (qRT-PCR), Western blotting, and immunostaining in human renal clear cell carcinoma cell line Caki-2. The obtained results allow further understanding of the interaction between GMFB and the TME in KIRC and thus provide a novel strategy for GMFB-based KIRC immunotherapies.

## Methods

### Data Collection and Differential Expression Analysis

RNA sequencing (RNA-seq), somatic mutations, and related clinical data were downloaded from TCGA (https://portal.gdc.cancer.gov/), containing 11,069 samples from 33 types of cancer (25). RNA sequence data from 539 patients with KIRC and 72 normal tissues were also downloaded from TCGA database. RNA-seq data and patient clinical information (Workflow Type: HTSeq-FPKM) were acquired using UCSC Xena (https://xena.ucsc.edu/), an online tool for the exploration of gene expression and clinical and phenotypic data. UALCAN (http://ualcan.path.uab.edu) (26) and the Human Protein Atlas (HPA) (https://www.Proteinatlas.org/) (27) were used to analyze GMFB protein expression patterns and immunohistochemical results in normal kidney tissue and KIRC samples.

### Survival Analysis and the Relationship Between *Gmfb* Expression and Prognosis

Survival and clinical phenotype data were extracted for each sample downloaded from TCGA database. Overall survival (OS) and disease-free survival (DFS) were evaluated to determine the relationship between *Gmfb* expression and patient prognosis. The Kaplan–Meier curve and Cox regression analyses were performed to assess the association between *Gmfb* expression and OS in KIRC patients. Subsequently, multivariate analysis was employed to estimate whether *Gmfb* expression was an independent prognostic factor for survival in patients with KIRC.

Gene expression profiling interactive analysis (GEPIA) (http://gepia2.cancer-pku.cn/#index) was used to confirm the correlation between *Gmfb* expression and clinicopathological information in multiple cancer types (28).

### Enrichment Analysis

The top 2,000 upregulated and downregulated differentially expressed genes (DEGs) between the high and low *Gmfb* expression groups, defined by Spearman’s rho above 0.30, were used for enrichment analyses. Pathways within the Kyoto Encyclopedia of Genes and Genomes (KEGG) and Gene Ontology (GO) terms were assessed for enrichment using the KEGG Orthology-Based Annotation System (KOBAS) platform (http://kobas.cbi.pku.edu.cn/) (29–32) and Metascape (https://metascape.org/gp/index.html#/main/step1) (33, 34).

### Assessment of Immune Infiltration

CIBERSORTx (http://cibersort.stanford.edu/) is a deconvolution algorithm that predicts the cell type components of intricate tissues based on normalized gene expression profiles; its results are usually in agreement with true estimates for many types of cancer (35). As so, CIBERSORTx was used to evaluate the relative proportions of 22 infiltrating immune cell subtypes in KIRC and LIHC (35). The percentages of the 22 immune cell subtypes derived from CIBERSORTx were regarded as a correction at p < 0.05 (36). The relationship between the expression of 22 subtypes of tumor-infiltrating immune cells (TIICs) and the survival of KIRC and LIHC patients was conducted using GEPIA2021 (http://gepia2021.cancer-pku.cns/) (37). The results of the survival analysis were considered statistically significant at p < 0.05.

### Protein–Protein Interaction Network Construction and Hub Gene Screening

Protein–protein interaction (PPI) networks were constructed using the Search Tool for the Retrieval of Interacting Genes (STRING; http://string-db.org/) database (38) (version 11.5) and Metascape (http://metascape.org). Hub genes were identified using the CytoHubba plugin (39) in the Cytoscape software (40) (version 3.7.7). The maximal clique centrality (MCC) algorithm was employed to identify hub genes.

### Cell Lines

The human embryonic kidney cell line 293T purchased from American Type Culture Collection (ATCC) and Caki-2 purchased from the Fenghui Institute of Biotechnology were used in this study. The HEK293T and Caki-2 cells were separately maintained in Dulbecco’s modified Eagle’s medium (DMEM)/high glucose and DMEM/nutrient mixture F12, respectively, supplemented with 10% fetal bovine serum (FBS) (Cat. No. 10091148, Gibco, Waltham, MA, USA) and 1% penicillin/streptomycin (Cat. No. 60162ES76, Yeasen, Shanghai, China) at 37°C and 5% CO_2_.

### Cell Transfection

The three pairs of small interfering RNA (siRNA) sequences of human *Gmfb* are shown in **Supplementary Table 1A**. A scrambled sequence was used as a siRNA control. The pcDNA-GMFB plasmid and pcDNA3.1 were kindly provided by Wan Sun. Caki-2 cells were transfected using Lipofectamine 2000 (Cat. No. T101, Vazyme, Nanjing, China), according to the manufacturer’s instructions. After transfection for 12, 24, and 48 h, cells were collected for subsequent experiments.

### Western Blotting

Caki-2 cells were lysed in radioimmunoprecipitation assay buffer (Cat. No. P0013B, Beyotime, Jiangsu, China) with protease inhibitors (Thermo Fisher Scientific, Waltham, MA, USA) to extract whole-cell proteins. Protein concentration was quantified using a bicinchoninic acid (BCA) kit (Cat. No. 20201ES76, Yeasen, Shanghai, China). A sample (30 μg) of each protein was then separated by sodium dodecyl sulfate–polyacrylamide gel electrophoresis, transferred to a polyvinylidene fluoride membrane (Millipore, Burlington, MA, USA), and blocked with 5% bovine serum albumin (BSA) at room temperature for 1 h. After incubation overnight at 4°C with primary antibodies against GMFB (1:1,000; Cat. No. 10690-1-AP, ProteinTech, Rosemont, IL, USA), GMFG (1:1,000; Cat. No. D121824, Sangon Biotech, Shanghai, China), p21 (1:1,000; Cat. No. 10355-1-AP, ProteinTech, Rosemont, IL, USA), p27 (1:1,000; Cat. No. #3686, CST, Danvers, MA, USA), and actin (1:10,000; Cat. No. 20536-1-AP, ProteinTech, Rosemont, IL, USA), membranes were incubated with a secondary antibody for 2 h. The secondary antibody used was horseradish peroxidase (HRP)-conjugated goat anti-rabbit IgG (1:10,000; Cat. No. SA00001-2, ProteinTech, Rosemont, IL, USA). The proteins were visualized using enhanced chemiluminescence (ECL) (Cat. No. E412-02, Vazyme, Jiangsu, China). Tanon 5200S (Tanon Science & Technology, Shanghai, China) was used to evaluate the density of protein bands, and relative protein levels were quantified using ImageJ software (https://imagej.nih.gov/ij/).

### Quantitative Real-Time PCR

Total RNA was extracted with TRIzol reagent (Cat. No. R401-01, Vazyme) and reverse transcribed into cDNA using the Revert Aid Reverse Transcription System (Takara, Dalian, China) according to the manufacturer’s protocol. PrimeScript RT polymerase (Cat. No. RR036A, Takara, Dalian, China) and SYBR Green Master Mix (Cat. No. FP205-02, Tiangen Biotech, Beijing, China) were used for conducting qRT-PCR on a LightCycler 96 Detection System (Bio-Rad Laboratories, Hercules, CA, USA). Data were analyzed using the 2^−ΔΔCt^ method (24). Oligos were synthesized by Sangon Biotech. The oligo sequences are shown in **Supplementary Table 1B**.

### Immunofluorescence

Immunofluorescence analysis was performed to detect the colocalization of GMFB and Arp2/3 complex. 293T and Caki-2 cells were fixed with 4% paraformaldehyde for 15 min, permeabilized with 0.3% Triton X-100 in phosphate-buffered saline (PBS) for 30 min, blocked with 3% BSA for 1 h, and incubated with primary antibodies against GMFB (1:200 Cat. No. 10690-1-AP, 60062-1-Ig, ProteinTech, Rosemont, IL, USA), Arp2 (1:50; Cat. No. D260865, Sangon Biotech, Shanghai, China), and Arp3 (1:50; Cat. No. sc-48344, Santa Cruz, Dallas, TX, USA) overnight with immunofluorescence buffer at 4°C. Cells were then incubated with secondary antibodies (Invitrogen, Waltham, MA, USA), goat anti-mouse IgG(H+L) cross-adsorbed secondary antibody, Alexa Fluor 488 (Cat. No. A-11001); goat anti-mouse IgG(H+L) cross-adsorbed secondary antibody, Alexa Fluor 555 (Cat. No. A-21422); goat anti-rabbit IgG(H+L) cross-adsorbed secondary antibody, Alexa Fluor 488 (Cat. No. A-11008); and goat anti-rabbit IgG(H+L) cross-adsorbed secondary antibody, Alexa Fluor 555 (Cat. No. A-21428) for 1 h at room temperature. After three washes for 10 min each with 1× PBS, cell sections were counterstained and mounted with DAPI Fluoromount-G (Cat. No. D9542, Sigma-Aldrich, St. Louis, MO, USA) and examined under a confocal microscope (Leica, Wetzlar, Germany).

### Determination of Cell Viability

Caki-2 cells were transfected with siRNA and pcDNA-GMFB using Lipo2000 (Cat. No. T101-01, Vazyme, Jiangsu, China). At 12, 24, and 48 h post-transfection, cell viability was tested using a Cell Counting Kit-8 (Cat. No. C0037, Beyotime, Jiangsu, China), according to the manufacturer’s instructions. This experiment was repeated four times.

### Measurement of Mitochondrial Membrane Potential

Caki-2 cells were transfected with an empty vector or *Gmfb* overexpression (OE) plasmid. JC-1 staining (Cat. No. C2003S, Beyotime, Jiangsu, China) was performed to determine mitochondrial membrane potential (MMP), according to the manufacturer’s instructions. Cell visualization was performed using fluorescence microscopy (Leica, Wetzlar, Germany).

### Wound Healing Assay

Caki-2 cells were seeded in six-well plates and grown to 80%–90% confluence. Confluent cell monolayers were scratched using a sterile 200-µl pipette tip and rinsed with PBS to remove scratched cells according to our previous work (24). Wound closure was observed after 24 h. The wound closure rate was calculated as follows: wound closure (%) = (the area of initial wound − the area of the final wound)/area of initial wound × 100.

### Transwell Migration and Invasion Assays

The migration and invasion abilities of cells were assessed using transwell migration (without Matrigel) and invasion (with Matrigel) assays in 24 wells according to our previous work (24). Cell suspensions in an FBS-free medium were added to the upper transwell chambers (24-well, 8-μm pores; BD, Franklin Lakes, NJ, USA), whereas 600 µl of the medium was supplemented with 10% FBS and added to the lower transwell chambers. The cell count after positive staining with crystal violet indicated migration or invasion.

## Results

### Clinical Characteristics of Kidney Renal Clear Cell Carcinoma Patients

Detailed information on the clinical and gene expression data of the downloaded 539 samples is presented in **Table 1**. The median age of participants was 61 years. Among the 539 patients, 353 were male (65.5%) and 186 were female (34.5%). The 269 samples of participants under the age of 60 accounted for 49.9% of all samples. The topographic distribution included 51.6% T1 (n = 278), 13.2% T2 (n = 71), 33.2% T3 (n = 179), and 2% T4 (n = 11) cases. Only 16 patients (6.2%) showed lymph node involvement, while 78 cases (15.4%) had distant metastases. Most patients (467, 87.8%) were white.

**Table 1:** Clinical characteristics of the kidney renal clear cell carcinoma patients.

Characteristic	Levels	Overall
n		539
T stage, n (%)	T1	278 (51.6%)
	T2	71 (13.2%)
	T3	179 (33.2%)
	T4	11 (2%)
N stage, n (%)	N0	241 (93.8%)
	N1	16 (6.2%)
M stage, n (%)	M0	428 (84.6%)
	M1	78 (15.4%)
Pathologic stage, n (%)	Stage I	272 (50.7%)
	Stage II	59 (11%)
	Stage III	123 (22.9%)
	Stage IV	82 (15.3%)
Gender, n (%)	Female	186 (34.5%)
	Male	353 (65.5%)
Race, n (%)	Asian	8 (1.5%)
	Black or African American	57 (10.7%)
	White	467 (87.8%)
Age, n (%)	≤60	269 (49.9%)
	>60	270 (50.1%)
Histologic grade, n (%)	G1	14 (2.6%)
	G2	235 (44.3%)
	G3	207 (39%)
	G4	75 (14.1%)
OS event, n (%)	Alive	366 (67.9%)
	Dead	173 (32.1%)
Age, median (IQR)		61 (52, 70)

OS, overall survival; IQR, interquartile range.

### Survival Atlas of *Gmfb* in Multiple Cancer Types and Differential Expression of GMFB Between Tumor and Normal Tissue Samples

**Figure 1:**
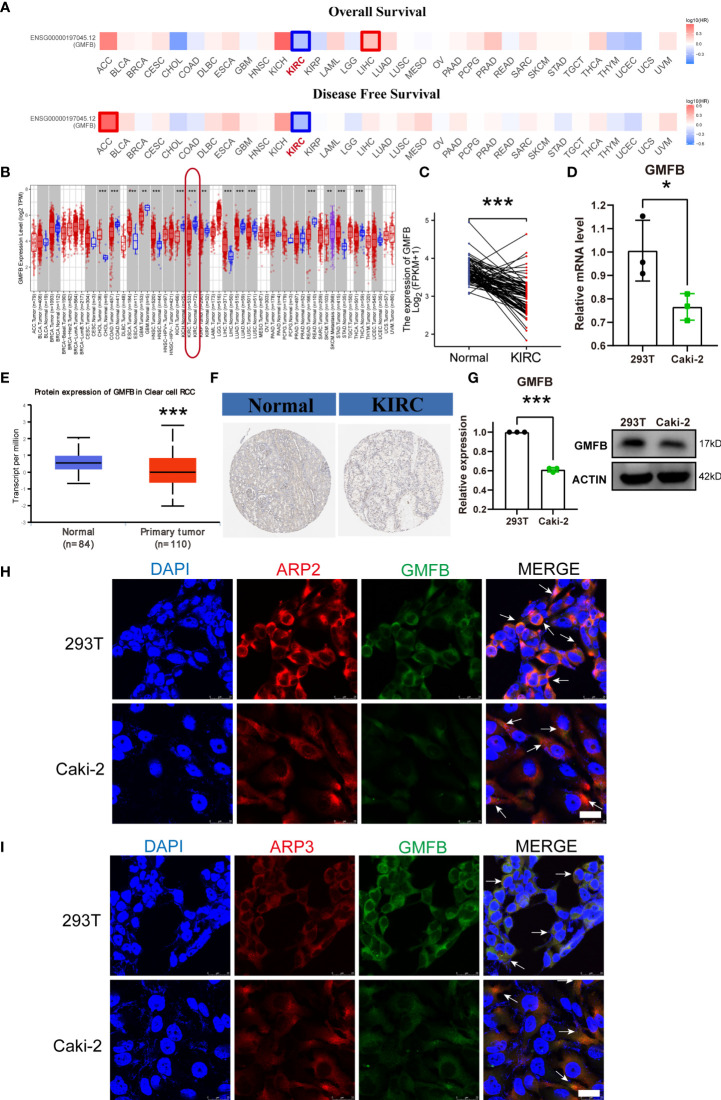
Survival map and differential expression of the mRNA and protein level of GMFB in pan-cancer and KIRC. **(A)** Comparing the survival contribution of GMFB in pan-cancer, estimated using Mantel–Cox test, including overall survival (OS) and disease-free survival (DFS). Tumor types with significant differences (p-value adjustment <0.05) were highlighted. HR, hazard ratio. **(B)** Comparison expression of *Gmfb* mRNA level between tumor and normal tissues (pair samples). **(C)** Comparison expression of *Gmfb* mRNA level between tumor and normal tissues (unpaired samples). **(D)** Comparison expression of *Gmfb* mRNA level between HEK293T and Caki-2 cells tested by RT-qPCR. **(E)** Comparison of GMFB protein level expression between normal and KIRC tumor tissues (unpaired samples). **(F)** Immunohistochemistry images in normal and KIRC tumor tissues. **(G)** Comparison expression of GMFB protein level between 293T and Caki-2 cells tested by Western blotting. **(H)** Colocalization of GMFB with Arp2 in 293T and Caki-2 cells. **(I)** Colocalization of GMFB co-localized with Arp3 in 293T and Caki-2 cells. Scale bar = 25 μm. White arrows indicate co-location. KIRC, kidney renal clear cell carcinoma. Error bars represent SD. *p < 0.05, **p < 0.01, ***p < 0.001.

The contribution of *Gmfb* to pan-cancer OS and DFS in the GEPIA2 database was compared using the Mantel–Cox test. The criterion for significant differences was a false discovery rate (FDR) <0.05, also known as p-value adjustment. OS analysis showed that *Gmfb* expression was negatively correlated with the hazard ratio (HR) of KIRC but positively correlated with the HR of LIHC in pan-cancers (**Figure 1A**). This was consistent with a previous report (24), in which *Gmfb* expression was negatively correlated with the HR of KIRC but positively correlated with the HR of adrenocortical carcinoma (ACC) (**Figure 1A**). Based on the correlation of *Gmfb* with OS and DFS in pan-cancer analysis, GMFB may be a promising novel prognostic factor for KIRC.

*Gmfb* expression levels were analyzed in pan-cancer tissues using TCGA dataset. Analysis of unpaired samples of 63 types of cancer tissues showed that *Gmfb* expression differed in 15 types of cancer tissues (**Figure 1B**), and it was downregulated in KIRC (p < 0.001) (**Figure 1B**). In the paired KIRC samples, the expression of *Gmfb* was also downregulated (p < 0.001) (**Figure 1C**). Protein expression analysis indicated that GMFB was weakly expressed in KIRC (p < 0.001) (**Figure 1E**). To verify GMFB expression at the protein level, we used the immunohistochemical results of KIRC provided by the HPA database (**Figure 1F**), which were consistent with those obtained from TCGA database. *Gmfb* was generally downregulated in renal cancer tissues.

*In vitro*, we confirmed that the *Gmfb* mRNA (**Figure 1D**) and protein (**Figure 1G**) levels were significantly decreased in Caki-2 cells when compared with HEK293T cells, which was consistent with the results of immunostaining of GMFB in HEK293T and Caki-2 cells (**Figures 1H–I**). We also detected the colocalization of GMFB and Arp2/3, which were known interaction partners of GMFB (**Figures 1H,I**), supporting that GMFB was an intracellular protein.

### Correlation of *Gmfb* Expression With Clinical Characteristics of Kidney Renal Clear Cell Carcinoma

The association between *Gmfb* expression and clinical features of KIRC patients is summarized in **Table 2**. T stage (p < 0.001), M stage (p < 0.004), primary therapy outcome (p = 0.016), pathologic stage (p < 0.001), gender (p = 0.002), histological grade (p < 0.001), and OS events (p < 0.001) were all significantly associated with *Gmfb* expression.

**Table 2:** Logistic analysis of the association between *Gmfb* expression and clinical characteristics.

Characteristics	Total (N)	Odds ratio (OR)	p-Value
T stage (T2 and T3 and T4 vs. T1)	530	0.503 (0.356–0.710)	<0.001
Pathologic stage (stage II and stage III and stage IV vs. stage I)	527	0.490 (0.346–0.692)	<0.001
N stage (N1 vs. N0)	255	0.299 (0.082–0.886)	0.041
M stage (M1 vs. M0)	498	0.468 (0.279–0.768)	0.003
Gender (male vs. female)	530	0.511 (0.355–0.734)	<0.001
Age (>60 vs. ≤60)	530	0.941 (0.669–1.323)	0.728
Hemoglobin (low and elevated vs. normal)	450	0.815 (0.559–1.188)	0.287

**Figure 2:**
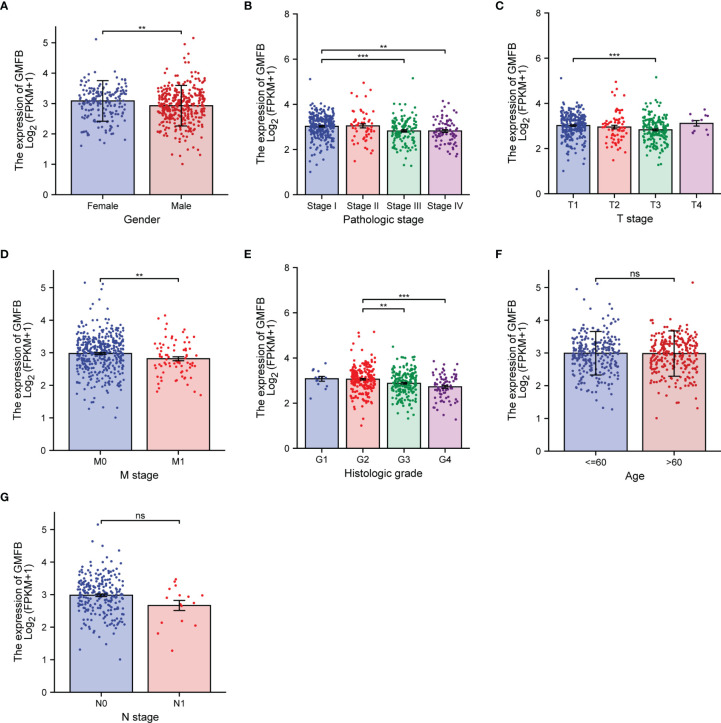
Association between the expression of *Gmfb* and the key clinicopathological characteristics of kidney renal clear cell carcinoma (KIRC). **(A)** Gender. **(B)** Pathologic grade. **(C)** T stage. **(D)** M stage. **(E)** Histologic grade. **(F)** Age. **(G)** N stage. **p < 0.01, ***p < 0.001, ns not significant.

The differential expression of *Gmfb* was examined according to age, gender, pathologic stage, T classification, N classification, M classification, and histological grade (G) of KIRC patients (**Figure 2**). Expression of *Gmfb* was significantly downregulated in men compared to women (p = 0.004) (**Figure 2A**); it was also significantly associated with pathologic stage (stage IV compared to stage I, p = 0.002; stage III compared to stage I, p < 0.001) (**Figure 2B**), T stage (T3 compared to T1, p < 0.001) (**Figure 2C**), M stage (M1 compared to M0, p = 0.007) (**Figure 2D**), and histological grade (G4 compared to G2, p < 0.001; G3 compared to G2, p = 0.003) (**Figure 2E**). There was no significant relationship between *Gmfb* expression and age (**Figure 2F**) or N classification (**Figure 2G**).

### Prognostic Value of *Gmfb* Across Kidney Renal Clear Cell Carcinoma

**Figure 3:**
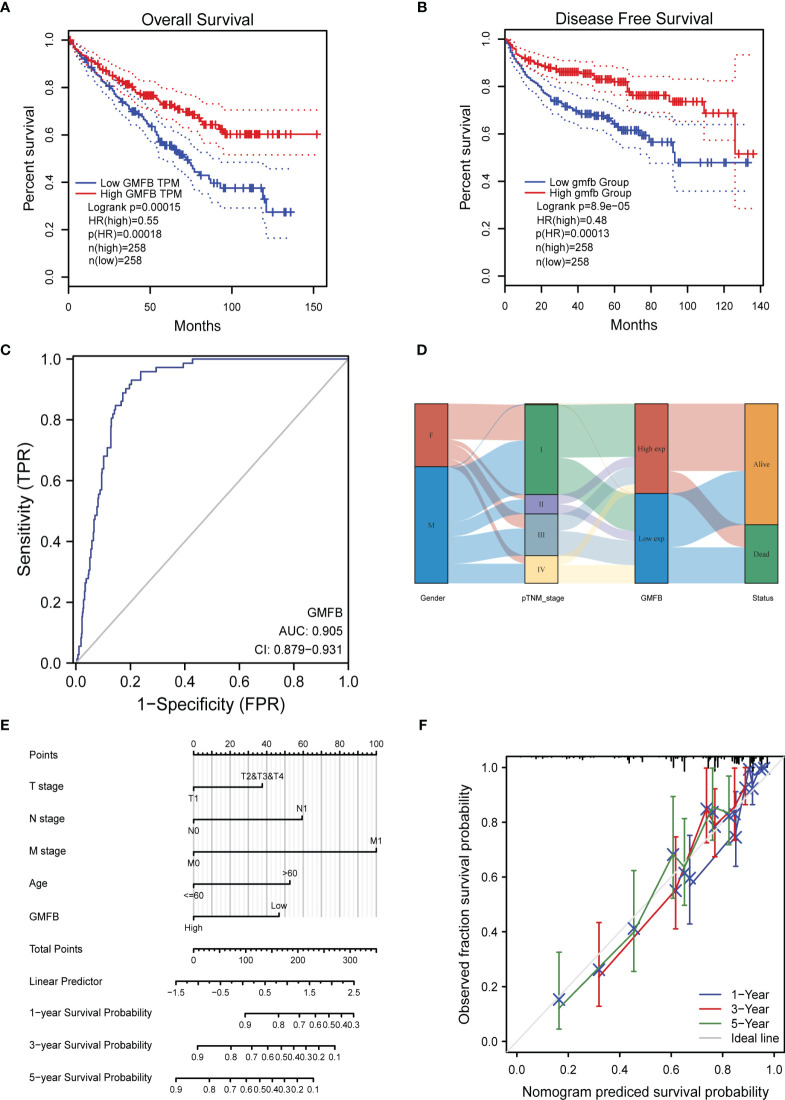
The prognostic value of *GMFB* expression in kidney renal clear cell carcinoma (KIRC). **(A)** Kaplan–Meier survival curves for the effect of *Gmfb* expression on overall survival. **(B)** Kaplan–Meier survival curves for the outcome of GMFB expression on disease-free survival. **(C)** Receiver operating characteristic analysis (ROC) curve of GMFB in KIRC. **(D)** *Gmfb* expression association with different clinical characteristics shown on the Sankey diagram. **(E)** The integrated *Gmfb* and other prognostic factors in KIRC from The Cancer Genome Atlas (TCGA) data by nomogram. **(F)** The calibration curve of the nomogram.

OS analysis showed that *Gmfb* was a low-risk gene in kidney tissue (p = 0.00018) (**Figure 3A**). The Kaplan–Meier DFS analysis also demonstrated that patients with high *Gmfb* expression levels survived longer than patients with low *Gmfb* expression (p = 0.00013) (**Figure 3B**). Cox OS analysis demonstrated that a significant prognostic difference existed between the high and low *Gmfb* expression groups in both univariate (HR 0.541, 95% CI, 0.397–0.736, p < 0.001) and multivariate (HR 0.578, 95% CI, 0.374–0.892; p = 0.013) analyses (**Table 3**). A receiver operating characteristic (ROC) curve was used to evaluate the prediction accuracy of intensity. The *Gmfb* score, in terms of the ROC curve, was 0.905 (**Figure 3C**), indicating that *Gmfb* was a very sensitive biomarker for KIRC.

**Table 3:** Univariate and multivariate Cox proportional hazard analyses of *Gmfb* expression and overall survival (OS) for patients with KIRC.

Characteristics	Total (N)	Univariate analysis		Multivariate analysis	
		Hazard ratio (95% CI)	p-Value	Hazard ratio (95% CI)	p-Value
T stage (T2 and T3 and T4 vs. T1)	530	2.872 (2.063–3.998)	**<0.001**	1.555 (0.936–2.584)	0.088
N stage (N1 vs. N0)	255	3.426 (1.818–6.456)	**<0.001**	2.006 (1.037–3.879)	**0.039**
M stage (M1 vs. M0)	498	4.333 (3.170–5.922)	**<0.001**	3.230 (1.994–5.232)	**<0.001**
Age (>60 vs. ≤60)	530	1.753 (1.290–2.383)	**<0.001**	1.856 (1.211–2.844)	**0.005**
Gender (male vs. female)	530	0.951 (0.697–1.296)	0.750		
GMFB (high vs. low)	530	0.541 (0.397–0.736)	**<0.001**	0.578 (0.374–0.892)	**0.013**

KIRC, kidney renal clear cell carcinoma. Bold values, p < 0,05

The Sankey diagram further demonstrated the association between age, gender, *Gmfb* expression, and clinical status (**Figure 3D**). Each column represents a characteristic (gender; T, N, and M stages; and expression of *Gmfb*; different colors represent the various states of these characteristics, and lines represent the distribution of the same sample in other characteristic variables). As shown in the diagram, most female and male patients progressed to stage I. Most patients with stage I disease had high *Gmfb* expression and a high survival rate. Conversely, patients with more severe diseases tended to have lower *Gmfb* expression and lower survival rates. A nomogram of OS was constructed to integrate *Gmfb* expression and some prognostic factors, including T, N, and M classifications and age (**Figure 3E**). A higher nomogram score represented worse predictive probability. The calibration curve assessed the performance of the nomogram for *Gmfb*, and the C-index for OS was 0.755 (**Figure 3F**). In conclusion, this nomogram is a good model for predicting the survival of patients with KIRC.

### The Effects of Overexpression of *Gmfb* in Caki-2 Cells on Viability, Proliferation, Invasion, and Mitochondrial Membrane Potential

**Figure 4:**
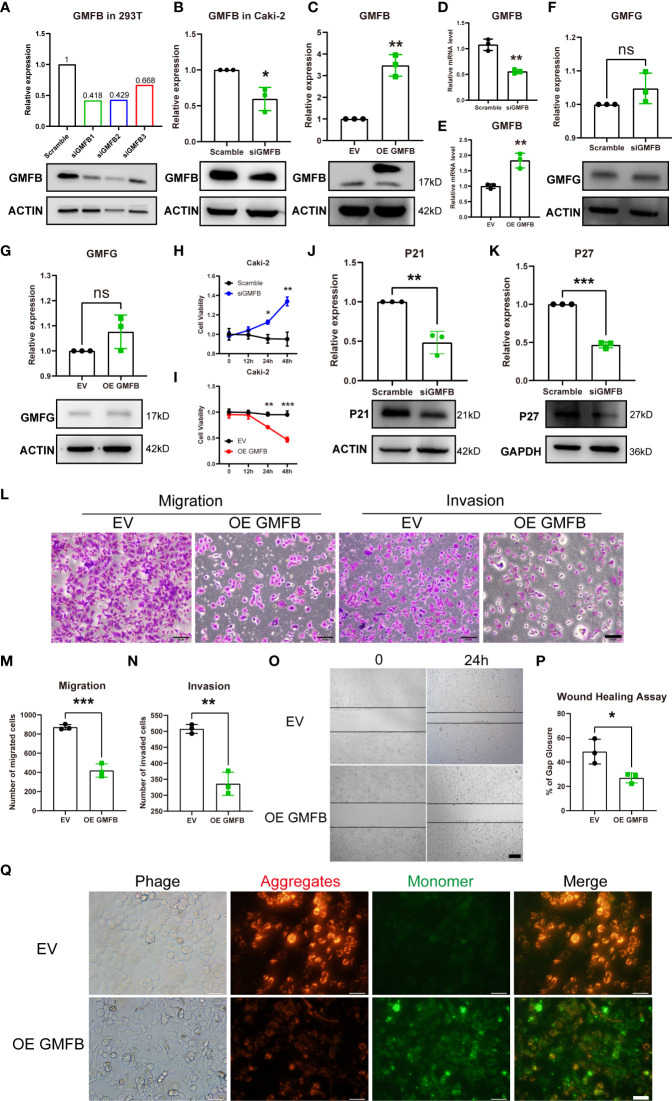
The effects of *Gmfb* knockdown and *Gmfb* overexpression on proliferation, migration, and invasion in Caki-2 cells. **(A)** The knockdown efficiency of three pairs of *siGmfb* was tested by Western blotting in HEK 293T cells. **(B–E)** GMFB silencing RNA and overexpression *Gmfb* plasmid efficiency were tested by Western blotting and RT-qPCR in Caki-2 cells. **(F, G)** GMFG protein level was tested by Western blotting after Caki-2 cells were transferred with siRNA and overexpression plasmid of GMFB. **(H, I)** Cell viabilities were determined after Caki-2 cells were transferred with siRNA and overexpression plasmid of *Gmfb* by CCK8 assay at 12, 24, and 48(h) **(J, K)** The extent of the cell cycle was further determined by CDKN1A (p21) and CDKN1B (p27) using Western blotting. **(L)** Transwell migration and invasion assays were used to examine the migration and invasion ability of Caki-2 cells. Scale bar = 50 μm. **(M, N)** Quantification of migration and invasion assays. **(O)** Image of the scratch assay. Scale bar = 100 μm. **(P)** Quantification of the scratch assay. **(Q)** Fluorescence microscopy imaging of Caki-2 cells stained with JC-1. Scale bar = 50 μm. Error bars represent SD. *p < 0.05, **p < 0.01, ***p < 0.001.

To clarify the association between *Gmfb* expression and cell viability in KIRC, the effects of *Gmfb* OE and knockdown (KD) on the viability of Caki-2 cells were examined. First, we designed three siRNA sequences for *Gmfb*, and KD efficiency was determined in HEK293T by Western blotting. As shown in **Figure 4A**, the siRNA1 with the highest KD efficiency in HEK 293T cells was used for the subsequent experiment (**Figure 4A**). Then, *Gmfb* KD and OE in Caki-2 cells were confirmed by Western blotting (**Figures 4B, C**) and qRT-PCR (**Figures 4D, E**). In parallel, the expression of GMFG protein, a homolog of GMFB, remained unchanged after transfection with *siGmfb* or Gmfb OE plasmid, indicating that *Gmfb* KD specificity was reasonable (**Figures 4F, G**). After transfection with siRNA and OE plasmids at 12, 24, and 48 h, a significant increase in viability after *Gmfb* KD was obtained at 24 h (p < 0.05) and 48 h (p < 0.01) (**Figure 4H**), while a significant decrease in viability after *Gmfb* OE was observed at 24 h (p < 0.01) and 48 h (p < 0.001) (**Figure 4I**). These results indicated that GMFB expression level was negatively correlated with tumor cell viability. Moreover, we investigate the effect of altered *Gmfb* expression on the Caki-2 cell cycle. At 48 h post-transfection of *siGmfb* in Caki-2 cells, the expression levels of p21 (CDKN1A) and p27 (CDKN1B) significantly decreased when compared with those in the scramble group (**Figures 4J, K**). The downregulation of p21 and p27 was required for the cellular transition from quiescence to the proliferative state (41). *Gmfb* OE in Caki-2 cells led to a lower number of migrated cells than in the empty vector group (**Figures 4L, M**). Invasion assays showed that *Gmfb* OE inhibited Caki-2 cell invasion (**Figures 4L, N**) and significantly reduced wound closure (**Figures 4O, P**). Detection of MMP using the JC-1 assay revealed a shift from red to green fluorescence in *Gmfb* OE Caki-2 cells (**Figure 4Q**), indicating that a high level of GMFB led to MMP loss. Taken together, we proposed that *Gmfb* was a suppressor gene in KIRC.

### Functional Enrichment Analysis of *Gmfb* in Kidney Renal Clear Cell Carcinoma Patients

**Figure 5:**
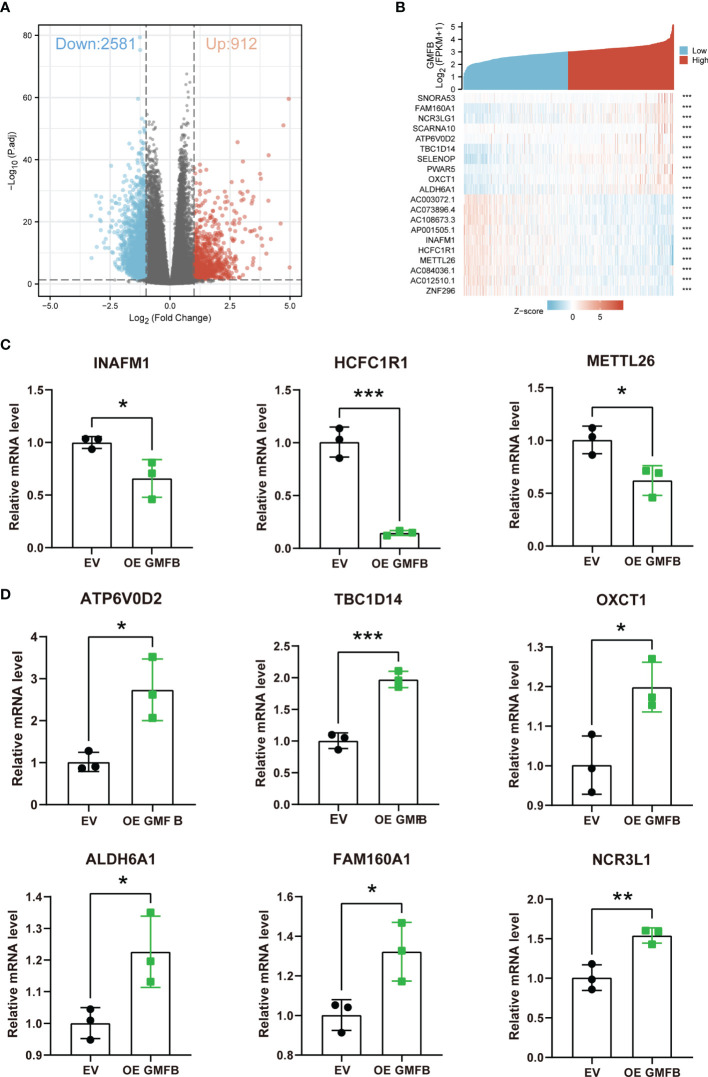
The identification of DEGs. **(A)** Volcano plot of differentially expressed genes (DEGs). **(B)** Heatmap of DEGs. **(C)** Downregulated DEGs (INAFM1, HCFC1R1, and METTL26) were determined by RT-qPCR. **(D)** Upregulated DEGs (ATP6V0D2, TBC1D14, OXCT1, ALDH6A1, FAM160A1, and NCR3LG1) were determined by RT-qPCR. Error bars represent SD. *p < 0.05, **p < 0.01, ***p < 0.001.

To elucidate the molecular mechanism of GMFB in KIRC, *Gmfb* co-expressed DEGs were used for functional enrichment analysis. The 3493 DEGs identified based on |log2 fold-change| > 1 (adjusted p < 0.05) corresponded to 912 upregulated genes and 2,581 downregulated genes. Detailed information on the DEGs is presented in **Supplementary Table 2**. DEG expression is shown in the volcano plot (**Figure 5A**), and the heatmap exhibits a corresponding hierarchical clustering analysis (**Figure 5B**). The details of the top 10 DEGs with upregulation and downregulation are listed in **Table 4** and **Supplementary Table 3**, respectively. Most of these DEGs have not been reported in previous KIRC studies, except for ALDH6A1 and NCR3LG1. Six DEGs with unknown biological functions and three non-coding RNAs were not identified, including AC003072.1, AC073896.4, AC108673.3, AP001505.1, AC012510.1, AC084036.1, PWAR5, SCARNA10, and SNORA53. Among these, the mRNA levels of seven upregulated and four downregulated DEGs with known functions were further validated by qRT-PCR. Due to the very high cycle threshold values for ZNF196 and SELENOP (38 to 39), we did not redesign the primer for the validation experiment. Consistent with the results of the meta-analysis, INAFM1, HCFC1R1, and METTL26 were downregulated, and ATP6V0D2, TBC1D14, OXCT1, ALDH6A1, FAM160A1, and NCR3LG1 were upregulated in *Gmfb* OE Caki-2 cells (**Figures 5C, D**). The details of the nine validated DEGs are listed in **Table 4**, and the remaining 11 DEGs that were not validated are provided in **Supplementary Table 3**.

**Table 4:** The significant DEGs with validation.

Gene symbol	p adj	Gene ID	Published role in KIRC	Function	Correlation with GMFB in meta-analysis	Validation in Caki-2
**INAFM1**	6.27E−53	255783	NOT	Predicted to be an integral component of membrane	Negative	Negative
**HCFC1R1**	8.32E−51	54985	NOT	Regulating HCFC1 activity by modulating its subcellular localization	Negative	Negative
**METTL26**	3.24E−50	84326	NOT	Diseases associated with METTL26 include anauxetic dysplasia 1	Negative	Negative
**ATP6V0D2**	4.02E−40	245972	NOT	Predicted to be involved in vacuolar acidification and vacuolar transport. Located in the apical plasma membrane. Part of vacuolar proton-transporting V-type ATPase complex	Positive	Positive
**TBC1D14**	3.95E−39	57533	NOT	Enabling protein kinase binding activity. Involved in negative regulation of autophagy, recycling endosome to Golgi transport, and regulation of autophagosome assembly	Positive	Positive
**OXCT1**	4.44E−37	5019	NOT	Key enzyme for ketone body catabolism. Transfers the CoA moiety from succinate to acetoacetate	Positive	Positive
**ALDH6A1**	2.68E−36	4329	PMID:33686951 PMID:32737333 PMID:30793530	Playing a role in valine and pyrimidine metabolism. Binds fatty acyl-CoA	Positive	Positive
**FAM160A1**	3.20E−33	729830	NOT	Involved in protein localization to perinuclear region of cytoplasm	Positive	Positive
**NCR3LG1**	4.93E−33	374383	PMID:31921143	Interaction of B7H6 with NK p30 results in natural killer (NK) cell activation and cytotoxicity	Positive	Positive
**ZNF296**	1.04E−46	162979	NOT	Enabling sequence-specific double-stranded DNA binding activity	Negative	NA
**SELENOP**	5.77E−38	6414	NOT	Might be responsible for some of the extracellular antioxidant defense properties of selenium or might be involved in the transport of selenium	Positive	NA

DEGs, differentially expressed genes; KIRC, kidney renal clear cell carcinoma. NA, Not Available

**Figure 6:**
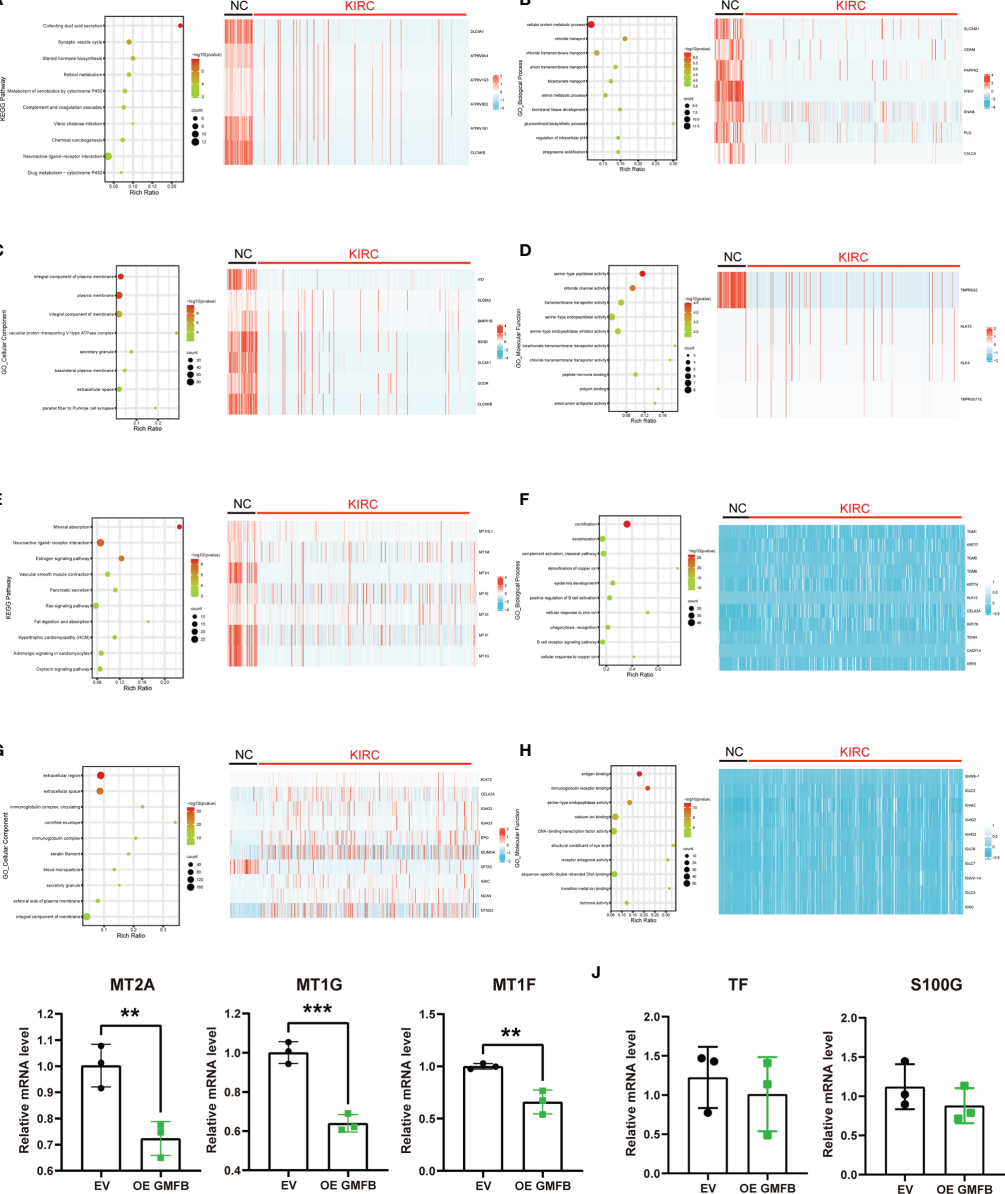
Differentially expressed genes with *Gmfb* in significant Kyoto Encyclopedia of Genes and Genomes (KEGG) pathway and Gene Ontology (GO) term analyses in kidney renal clear cell carcinoma (KIRC). **(A)** Significant KEGG pathways of 958 upregulated differentially expressed genes (DEGs) and the heatmap of genes in the first pathway collecting duct acid secretion. **(B)** Enriched GO-BP terms in 958 upregulated DEGs and the heatmap of genes in the first term cellular protein metabolic process. **(C)** Enriched GO-CC terms in 958 upregulated DEGs and the heatmap of genes in the first term integral component of the plasma membrane. **(D)** Enriched GO-MF terms in 958 upregulated DEGs and the heatmap of genes in the first term serine-type peptidase activity of **(G, E)** Significant KEGG pathways of 2,581 downregulated DEGs and the heatmap of genes in the first pathway mineral absorption. **(F)** Enriched GO-BP terms in 2,581 downregulated DEGs and the heatmap of genes in the first term cornification. **(G)** Enriched GO-CC terms in 2581 downregulated DEGs and the heatmap of genes in the first term extracellular region. **(H)** Enriched GO-MF terms in 2,581 downregulated DEGs and the heatmap of genes in the first term antigen binding. **(I, J)** The downregulated DEGs of mineral absorption were determined by RT-qPCR. **(I)** MT2A, MT1G, and MT1F had a significant difference. **(J)** TF and S100G had no significant difference. Error bars represent SD. **p < 0.01, ***p < 0.001.

The KEGG pathway and GO enrichment analyses using KOBAS are shown in **Figure 6**. The neuroactive ligand–receptor interaction ranked 1 in the top 10 DEGs enriched KEGG pathways, followed by collecting duct acid secretion, linoleic acid metabolism, alpha-linolenic acid metabolism, and mineral absorption (**Supplementary Figure 1A**). GO enrichment analysis showed that cornification, extracellular region, and serine-type endopeptidase activity were the most enriched terms in biological process (BP), cellular compartment (CC), and molecular function (MF) categories, respectively (**Supplementary Figure 1B** and **Supplementary Table 4**). The top ten enriched KEGG pathways, GO terms, and the heatmap of associated DEGs are shown in **Figures 6A–D** (upregulated DEGs) and **Figures 6E–H** (downregulated DEGs). Upregulated DEGs were most enriched in collecting duct acid secretion (KEGG pathway), cellular protein metabolic process (BP category of GO), an integral component of the plasma membrane (CC of GO), and serine-type endopeptidase activity (MF of GO) (**Figures 6A–D**). Downregulated DEGs were most enriched in mineral absorption (KEGG pathway), cornification (BP of GO), extracellular region (CC of GO), and antigen-binding (MF of GO) (**Figures 6E–H**). There was more overlap in the KEGG pathway and GO analyses between total DEGs and downregulated DEGs than between upregulated DEGs, indicating that the downregulated DEGs may play a more critical and dominant role in KIRC. To this end, we selected five genes involved in the mineral absorption pathway (MT2A, 1G, 1F, TF, and S100G) for qRT-PCR validation of the RNA-seq results. The qRT-PCR revealed that MT2A, MT1G, and MT1F were downregulated, whereas S100G and TF did not show a significant change in *Gmfb* OE Caki-2 cells (**Figures 6I, J**).

**Figure 7:**
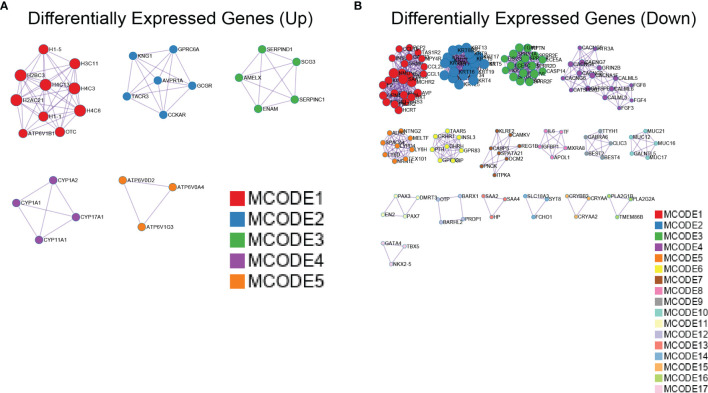
Protein–protein interaction (PPI) network of differentially expressed genes (DEGs) with *Gmfb* in kidney renal clear cell carcinoma (KIRC) by Metascape. **(A)** Upregulated DEGs. **(B)** Downregulated DEGs.

To clarify the links between terms, PPI network and MCODE component analyses of the top 2,000 DEGs (**Supplementary Figure 1C**), upregulated (**Figure 7A**) and downregulated (**Figure 7B**), were performed using Metascape. **Supplementary Table 5** provides detailed data. The PPI network showed that the predominant GO annotations of MCODE1 were cornified envelopes and keratinization in the total DEGs. Among the DEGs associated with upregulated *Gmfb*, GO annotations of MCODE1 were mainly linked with nucleosome assembly, nucleosome organization, and chromatin assembly. Among the DEGs related to downregulated *Gmfb*, GO annotations of MCODE1 were specifically associated with G-protein-coupled receptor (GPCR) downstream signaling, GPCR signaling, and GPCR ligand binding (**Supplementary Table 5**).

### Identification of Hub Genes Using STRING and Cytoscape

**Figure 8:**
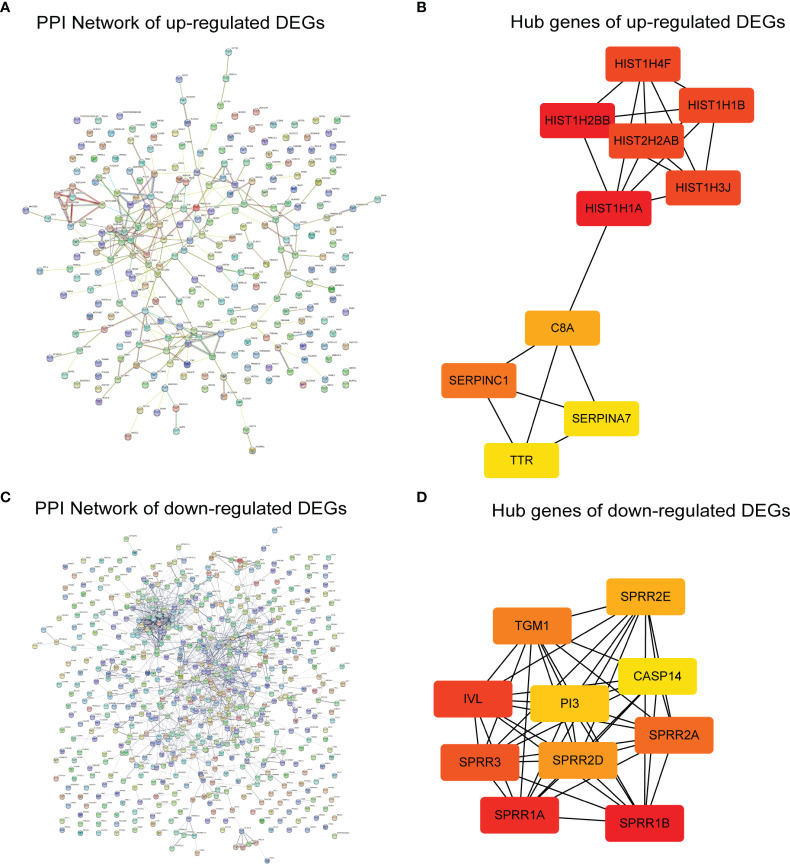
Identification of hub genes. **(A)** The protein–protein interaction (PPI) network was constructed with upregulated differentially expressed genes (DEGs). **(B)** The hub genes identified with Cytoscape for upregulated DEGs. **(C)** The PPI network was constructed with downregulated DEGs. **(D)** The hub genes identified with Cytoscape for downregulated DEGs.

Upregulated DEGs were projected separately into the human PPI network in the STRING database, and the hub genes were filtered using the MCC algorithm (**Figure 8**). The details of the top ten hub genes are shown in **Supplementary Table 6**. The top six upregulated hub genes (HIST1H2BB, HIST1H1A, HIST1H4F, HIST1H3J, HIST2H2AB, and HIST1H1B) (**Figure 8B**) belong to the histone cluster family and are mainly involved in nucleosome assembly, nucleosome organization, and chromatin assembly. The top ten downregulated hub genes included SPRR1B, SPRR1A, IVL, SPRR3, SPRR2A, TGM1, SPRR2D, SPRR2E, PI3, and CASP14 (**Figure 8D**). Except for CASP14 and TGM1, these genes were mainly associated with keratinization, and none has been reported in KIRC. These results suggest that GMFB may play a vital role in modulating nucleosome structure, cellular senescence, mitotic prophase, and keratinization in KIRC.

### Correlation Between *Gmfb* Expression, Immune Cell Infiltration, and Prognostic Value of Tumor-Infiltrating Immune Cells in Kidney Renal Clear Cell Carcinoma

**Figure 9:**
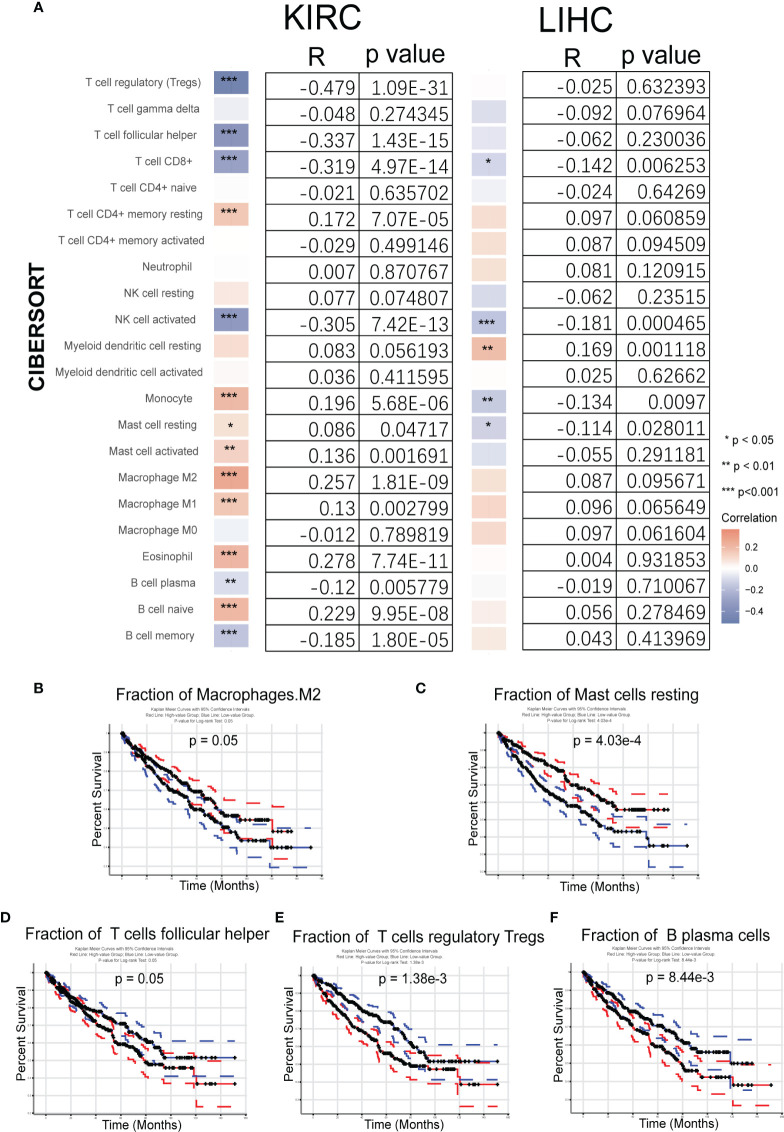
Analysis of the fraction of tumor-infiltrating immune cells (TIICs) in kidney renal clear cell carcinoma (KIRC) and liver hepatocellular carcinoma (LIHC). **(A)** Correlation between 22 tumor-infiltrating immune cells and *Gmfb* expression in KIRC and LIHC samples from The Cancer Genome Atlas (TCGA). **(B–F)** Kaplan–Meier survival curves for TIICs on overall survival. **(B)** Macrophages M2, **(C)** resting mast cells, **(D)** T help cells, **(E)** regulatory T cells (Tregs), and **(F)** plasma cells. *p < 0.05, **p < 0.01, ***p < 0.001

Based on the importance of TME, the relationship between immunity and *Gmfb* expression was further explored. Tumor RNA-seq data (retrieved from TCGA) of 33 different tumor patients and matched normal tissue samples from the Genomic Data Commons (GDC) website were downloaded to analyze the relationship between *Gmfb* expression and 22 types of TIICs. The heatmap of KIRC and LIHC analysis indicated *Gmfb* mRNA expression significantly correlated with TIICs (**Figure 9A**). The analysis by CIBERSORTx showed that the *Gmfb* mRNA expression positively correlated with the infiltration of T CD4+ memory resting cells (p = 0.00007, R = 0.172), monocytes (p = 5.68E−06, R = 0.196), mast resting cells (p = 0.047, R = 0.086), mast activated cells (p = 0.0017, R = 0.136), macrophage M2 cells (p = 1.81E−09, R = 0.257), macrophage M1 T cells (p = 0.0028, R = 0.13), eosinophil (p = 7.74E−11, R = 0.278), and B naïve cells (p = 9.95E−08, R = 0.229) but negatively correlated with the infiltration of regulatory T cells (Tregs) (p = 1.09E−31, R = −0.479), T follicular helper cells (TFH) (p = 1.43E−15, R = −0.337), T CD8+ cells (p = 4.97E−14, R = −0.319), natural killer activated cells (p = 7.42E−13, R = −0.305), B plasma cells (p = 0.0058, R = −0.12), and B memory cells (p = 0.00001, R = −0.185) (**Figure 9A**). However, our previous work showed that higher expression levels of *Gmfb* predicted poor prognostic outcomes in LIHC (26). To explore the possible mechanism underlying the differential effects of *Gmfb* on LIHC and KIRC, the correlation between *Gmfb* expression and TIIC in LIHC was analyzed. As shown in **Supplementary Figure 2**, the infiltration of B memory cells, mast activated cells, and neutrophils was significantly increased in LIHC (**Supplementary Figures 2A–B**) and was uniformly correlated with worse OS (**Supplementary Figures 2C–E**). None of these three immune cell types affected the OS of KIRC patients (**Supplementary Figure 3**). Moreover, the levels of B memory cells, mast activated cells, and neutrophils did not correlate significantly with *Gmfb* expression in the LIHC group (**Figure 9A**).

The association between TIICs and KIRC tumor tissues was examined. The relationship between the 22 TIICs and KIRC prognosis was investigated using GEPIA2021. The Kaplan–Meier survival curves (p < 0.05) are shown in **Figures 9B–F**, and further results are summarized in **Supplementary Figure 3**. The fractions of macrophages M2 cells (p = 0.05) and resting mast cells (p = 0.000403) were associated with improved survival (**Figures 9B, C**), but high levels of TFH cells (p = 0.05), Tregs (p = 0.00138), and B plasma cells (p = 0.00844) were associated with worse outcomes (**Figures 9D–F**).

Taken together, the higher expression of *Gmfb* in KIRC predicted a better prognosis, partially due to the optimal TME.

## Discussion

KIRC is a cancer with high prevalence and mortality (3). However, it can be diagnosed early and successfully treated with surgical or ablative strategies; up to a third of cases present with or develop metastases (42). Thus, a deeper understanding of the molecular mechanisms underlying KIRC is needed. The present study integrated different KIRC databases with suitable meta-analysis approaches, identified GMFB as a novel independent prognostic biomarker, positively correlated with OS and DFS in KIRC patients, and further dissected the possible contribution of GMFB-relevant TME to good clinical outcomes in KIRC patients.

Two studies have shown that GMFB is a prognostic biomarker for astrocytomas (43) and serous ovarian cancers (44). However, using GEPIA2.0 and TCGA in pan-cancer analysis, we failed to obtain consistent results with previous reports (data not shown), possibly due to dynamic updates of the cancer database with increasing sample data deposition. We previously found that *Gmfb* KD significantly inhibited the proliferation, migration, and invasion of Hep3B cells, and higher *Gmfb* expression predicted a poor prognosis in LIHC (24). In the present study, a completely distinct relationship between *Gmfb* and OS/DFS in KIRC was obtained; namely, higher expression of GMFB was associated with a better prognosis for KIRC patients (**Figure 3**). To confirm the meta-analysis results, *Gmfb* KD and OE were performed in Caki-2 cells. We found that *Gmfb* KD promoted cell viability partly due to the downregulation of p21 and p27 protein levels in Caki-2 cells (**Figures 4J, K**). The downregulation of p21 and p27 protein levels indicated the transition of the cell cycle from quiescent to proliferation (41). Moreover, *Gmfb* OE inhibited the proliferation, migration, and decreased MMP in Caki-2 cells, and when MMP was disturbed, reactive oxygen species caused the oxidation of mitochondrial pores (45). Taken together, the *in vitro* results support that high expression of GMFB is beneficial for KIRC patients’ survival. The distinct molecular mechanisms underlying GMFB in LIHC and KIRC merit further investigation.

To reveal the molecular mechanism underlying *Gmfb* effects in KIRC, we enriched the DEGs with *Gmfb* co-expression for bioinformatics analysis. KEGG pathway and GO analyses were performed for the top 2,000 downregulated and upregulated DEGs. We validated nine significant DEGs. Consistent with the results from the meta-analysis, INAFM1, HCFC1R1, and METTL26 were downregulated, and ATP6V0D2, TBC1D14, OXCT1, ALDH6A1, FAM160A1, and NCR3LG1 were upregulated in GMFB overexpressed Caki-2 cells (**Figures 5C, D**). The validated DEGs with GMFB co-expression may participate in multiple pathways in KIRC biology, including enhancing response in KIRC cell to NK cell (NCR3LG1) (46), negatively regulating autophagy (TBC1D14) (47), and being regulated by lactate (ATP6V0D2) (48) in TMEs.

In the upregulated pathways, collecting duct acid secretion was the most enriched, containing DEGs ATP6V0A4, ATP6V1G3, ATP6V0D2, ATP6V1B1 (vacuolar proton pump subunit), SLC4A1 (chloride/bicarbonate exchanger), and CLCNKB (voltage-gated chloride channels). Lower CLCNKB expression has been reported in renal cancer (49). We hypothesized that the upregulated collecting duct acid secretion might play a role in maintaining acid–base homeostasis in the renal tubule.

Mineral absorption, neuroactive ligand–receptor interaction, and estrogen signaling in the downregulated panel were the three most enriched pathways (**Figure 6E**). Metallothionein (MT) proteins, such as MT1A, B, E, G, F, H, 349 HL1, X, M, MT2A, TF, and S100G, were enriched in the mineral absorption pathway. MTs have a high content of cysteine residues that bind various heavy metals. In this family, MT1A, B, E, G, F, H, HL1, X, M, MT2A, TF, and S100G (vitamin D-dependent calcium-binding protein) are enriched in the mineral absorption pathway. The dysregulation of MTs in renal cell cancer has also been reported. The toxic metals cadmium and lead are associated with renal cell cancer progression (50). A decrease in MT levels in renal cell cancer has also been reported (51). In the present study, we conducted qRT-PCR to validate some of the mRNA levels of several MTs in Caki-2 cells. The results showed that MT2A, MT1G, and MT1F were downregulated, but S100G and TF did not show a significant change in *Gmfb* OE Caki-2 cells (**Figure 6I**). This indicates that low expression of *Gmfb* may lead to increased circulating levels of toxic heavy metals, thus contributing to the progression of KIRC. Currently, there are no relevant reports on S100G and TF in KIRC, and their role, therefore, remains unclear. Cornification, keratinization, and the classical complement activation pathway were significantly enriched in the BP category of GO. There were 23 shared genes between the BP terms cornification and keratinization (**Supplementary Table 7**). Only CK7 (52, 53) and AE1AE3 (54) cytokeratins have been examined in KIRC. The fundamental functions of other cornification-related genes remain elusive, probably implicating an alteration of epithelial and mesenchymal immunophenotypes for KIRC, which requires further investigation. KOBAS-based GO enrichment analysis revealed downregulated DEGs in the extracellular region, extracellular space, and Ig complex circulating GO-CC category (**Figure 6G**). Most genes in the extracellular region (**Supplementary Table 4**) encode MMPs, cytokines, chemokines, growth factors, and others. These results, combined with the DEGs enriched in GO-MF (antigen binding, Ig receptor binding, and serine-type endopeptidase activity) (**Figure 6H**), suggest cell–cell interactions.

Analyzing PPI networks is increasingly recognized as meaningful to characterize the underlying biology of genes associated with complex diseases (55). As shown in **Figure 8**, histone cluster family members were enriched in CytoHubba with upregulated DEGs (**Figure 8B**), and the formation of the cornified envelope was enriched in CytoHubba with downregulated DEGs (**Figure 8D**). The former is possibly linked to epigenetic regulation in KIRC. In contrast, the latter may contribute to metastasis by disrupting the formation of the cornified envelope, which requires further validation.

The actual context of KIRC is quite diverse due to intratumoral or intertumoral TME heterogeneity. The enriched pathways and GO terms, including extracellular region and neuroactive ligand–receptor binding, encouraged us to analyze the *Gmfb*-relevant TME.

**Figure 10:**
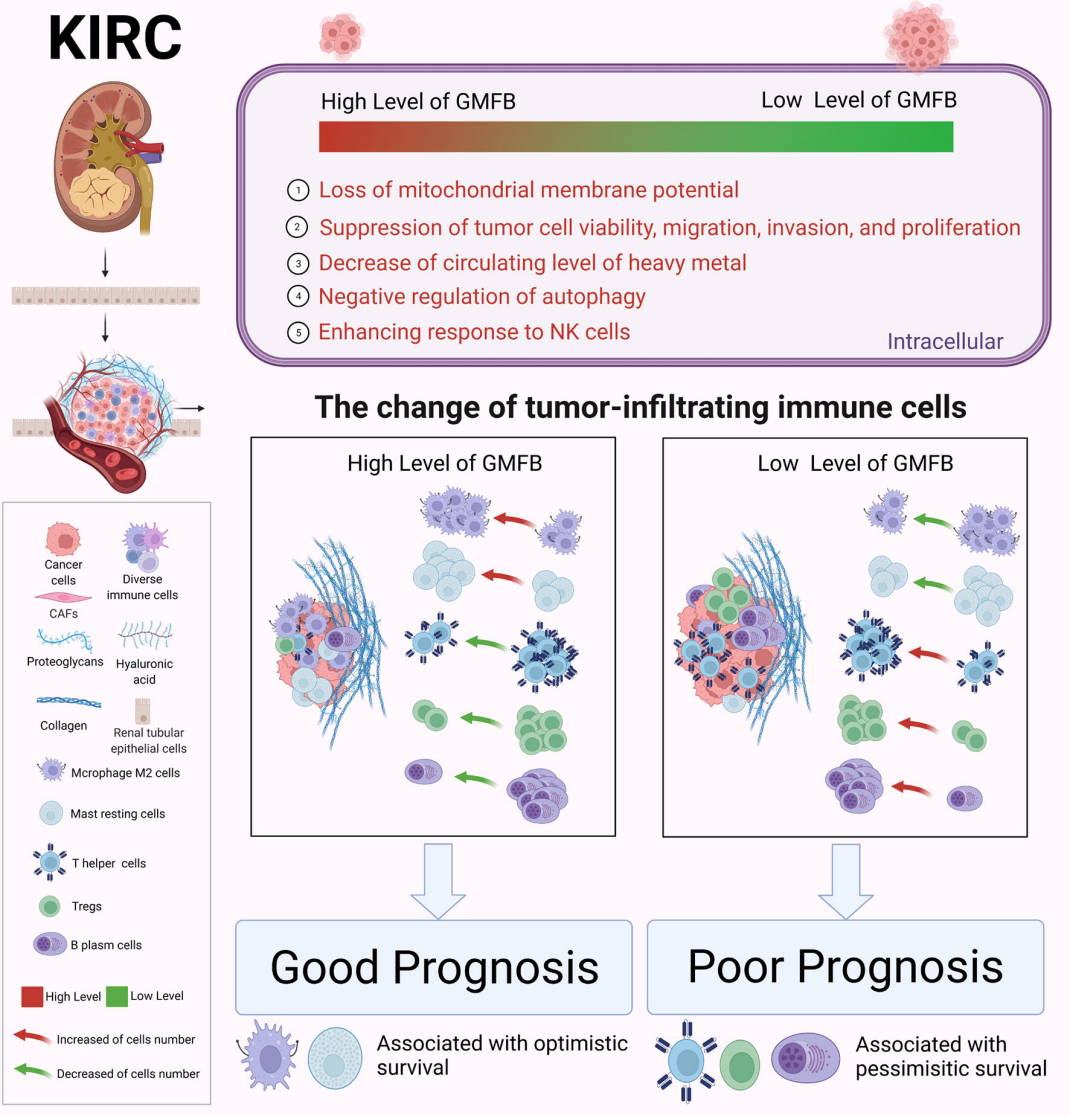
Schematic molecular mechanism of GMFB in the development of kidney renal clear cell carcinoma (KIRC). Created with BioRender.com.

TME is a complex ecosystem, and it plays a crucial function in cancer progression and response to immunotherapy (7, 56). The TME is composed of adaptive immune cells (T and B lymphocytes) and innate immune response cells (including dendritic cells, mast cells, macrophages, neutrophils, myeloid-derived suppressor cells, and natural killer cells) as well as cancer cells and stroma (i.e., endothelial cells, fibroblasts, pericytes, and mesenchymal cells) (56). Immune and inflammatory responses are strongly associated with survival outcomes in patients with KIRC. Zhang et al. recently revealed insights into the TME in KIRC using single-cell sequencing technology (57) and elucidated an active role for tumor epithelia in promoting immune cell infiltration. In contrast, the immune cell composition in the TME may impact clinical outcomes. Infiltration by CD8+ T cells is associated with a worse prognosis in KIRC (58). CCR4 has been identified as a TME target for renal cancer (59). In the present study, we further integrated the OS data obtained from GEPIA2021 with the results of CIBERSORTx. We found a significant association between GMFB-associated immune cells and OS in patients with KIRC. A significant correlation between GMFB expression and KIRC is shown in **Figure 9A**. GMFB expression was positively correlated with macrophage M2 cells and resting mast cells but negatively associated with T follicular helper cells, Tregs, and plasma cells (**Figure 9A**). In addition, high levels of M2 macrophages (p = 0.05) and resting mast cells (p = 0.000403) favored a better prognosis (**Figures 9B, C**), while high levels of TFH cells (p = 0.05), Tregs (p = 0.00138), and plasma cells (p = 0.00844) caused deleterious clinical outcomes in patients with KIRC (**Figures 9D–F**). We summarized the role of *Gmfb* and *Gmfb*-related TME in KIRC in **Figure 10**.

The *Gmfb*-related TME in LICH was analyzed. Unexpectedly, the *Gmfb*-related TME in LIHC was quite different from that in KIRC (**Supplementary Figure 2**). Thus, we speculated that the distinct effects of *Gmfb* expression on the OS of LIHC and KIRC patients might be due to different immune cell infiltration.

This study had several limitations. The first one was mainly based on bioinformatics analysis, especially datasets from bulk RNA-seq, and lacked validation experiments with human KIRC samples. Secondly, the *Gmfb*-related TME also needed to be validated with human KIRC samples. The critical molecular signatures identified in KIRC merit further clinical investigation.

In conclusion, our study demonstrated that GMFB could be considered a novel independent prognostic biomarker for KIRC. Modulation of the TME in KIRC by GMFB intervention may represent a novel immunotherapeutic strategy for KIRC and probably improve clinical outcomes.

## Data Availability Statement

The datasets presented in this study can be found in online repositories. The names of the repository/repositories and accession number(s) can be found in the article/**Supplementary Material**.

## Author Contributions

LL, GX, and JW conceived, developed, and mentored the project. TZ designed the study, performed the experiments, and wrote the manuscript. TW performed part of the data analysis and interpreted the experimental data. ZF performed part of the data analysis. FG, JZ, CJ, HT, JX, HC, and QO provided technical support and analyzed the data. All authors read and approved the final manuscript.

## Funding

This paper was supported by the Ministry of Science and Technology of China (2020YFA0113101), the National Natural Science Foundation of China (32070719, 81770942), and the Fundamental Research Funds for the Central Universities (22120220009).

## Conflict of Interest

The authors declare that the research was conducted in the absence of any commercial or financial relationships that could be construed as a potential conflict of interest.

## Publisher’s Note

All claims expressed in this article are solely those of the authors and do not necessarily represent those of their affiliated organizations, or those of the publisher, the editors and the reviewers. Any product that may be evaluated in this article, or claim that may be made by its manufacturer, is not guaranteed or endorsed by the publisher.
