# Supplementary material for: Glia Maturation Factor β as a Novel Independent Prognostic Biomarker and Potential Therapeutic Target of Kidney Renal Clear Cell Carcinoma
Source: Front Oncol. 2022 Jul 4;12:880100. doi: 10.3389/fonc.2022.880100 (PMC9292986; doi:10.3389/fonc.2022.880100)
Supplement: Supplementary file 1 [file DataSheet_1.zip › Supplementary Figure Legands.docx]

**Supplementary Figure1.** (a) Significant KEGG pathways of top 2000 total DEGs, p values are marked differently. (b) Enriched BP, CC, and MF GO terms in top 2000 total DEGs. (C) PPI network of DEGs with *GMFB* in KIRC by metascape.

**Supplementary Figure2.** The prognostic value of TIICs in LIHC. (A-B) B memory cells, Tregs, T gamma delta cells, macrophages.M0, myeloid dendritic resting cells, mast activated cells, and neutrophils are increased significantly in LIHC. Monocytes, macrophages.M2, mast resting cells are decreased considerably in LIHC. Higher fractions of B memory cells (C), mast activated cells (D), and neutrophils (E) are significantly correlated with worse overall survival. Kaplan Meier Curves with 95% Confidence Intervals. Red Line: High-value Group; Blue Line: Low-value Group.

**Supplementary Figure3.** Kaplan-Meier survival curves for TIICs on overall survival. (a) B naïve cells, (b) B memory cells, (c) Dendritic activated cells, (d) Dendritic resting cells, (e) Eosinophils, (f) Macrophage M0 cells, (g) Macrophage M1 cells, (h)Mast activated cells, (i) Monocytes, (j) Neutrophils, (k) NK activated cells, (l) NK resting cells, (m) T CD4+ activated cells, (n) T CD4+ resting cells, (o)T CD4+ naïve cells, (p) T CD8+ cells, (q) T gamma delta cells.
