# Supplementary figures and images for: Glia Maturation Factor β as a Novel Independent Prognostic Biomarker and Potential Therapeutic Target of Kidney Renal Clear Cell Carcinoma
Source: Front Oncol. 2022 Jul 4;12:880100. doi: 10.3389/fonc.2022.880100 (PMC9292986; doi:10.3389/fonc.2022.880100)

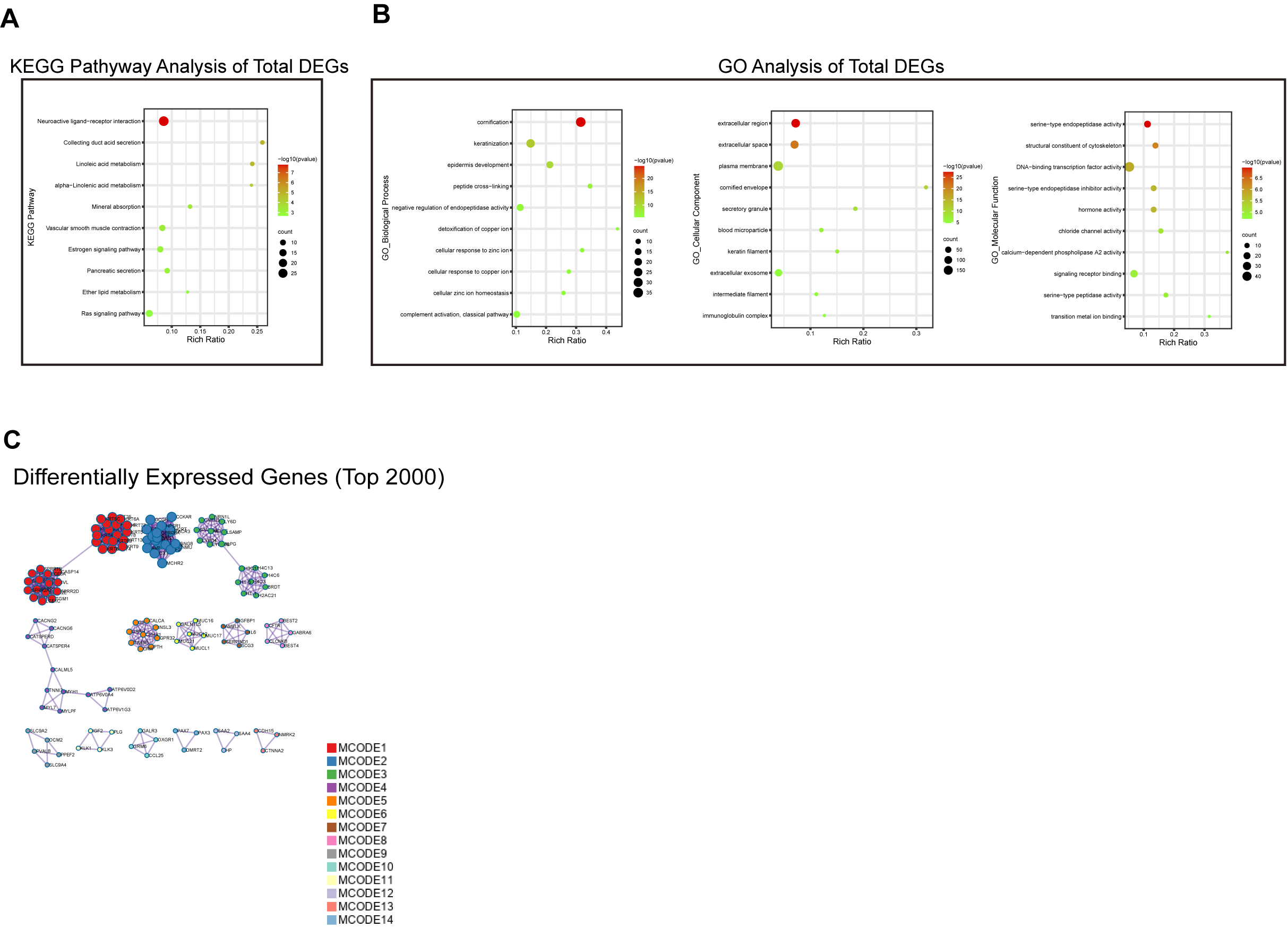

Supplement: Supplementary file 1 [file DataSheet_1.zip › Supplementary Figure1..tif]

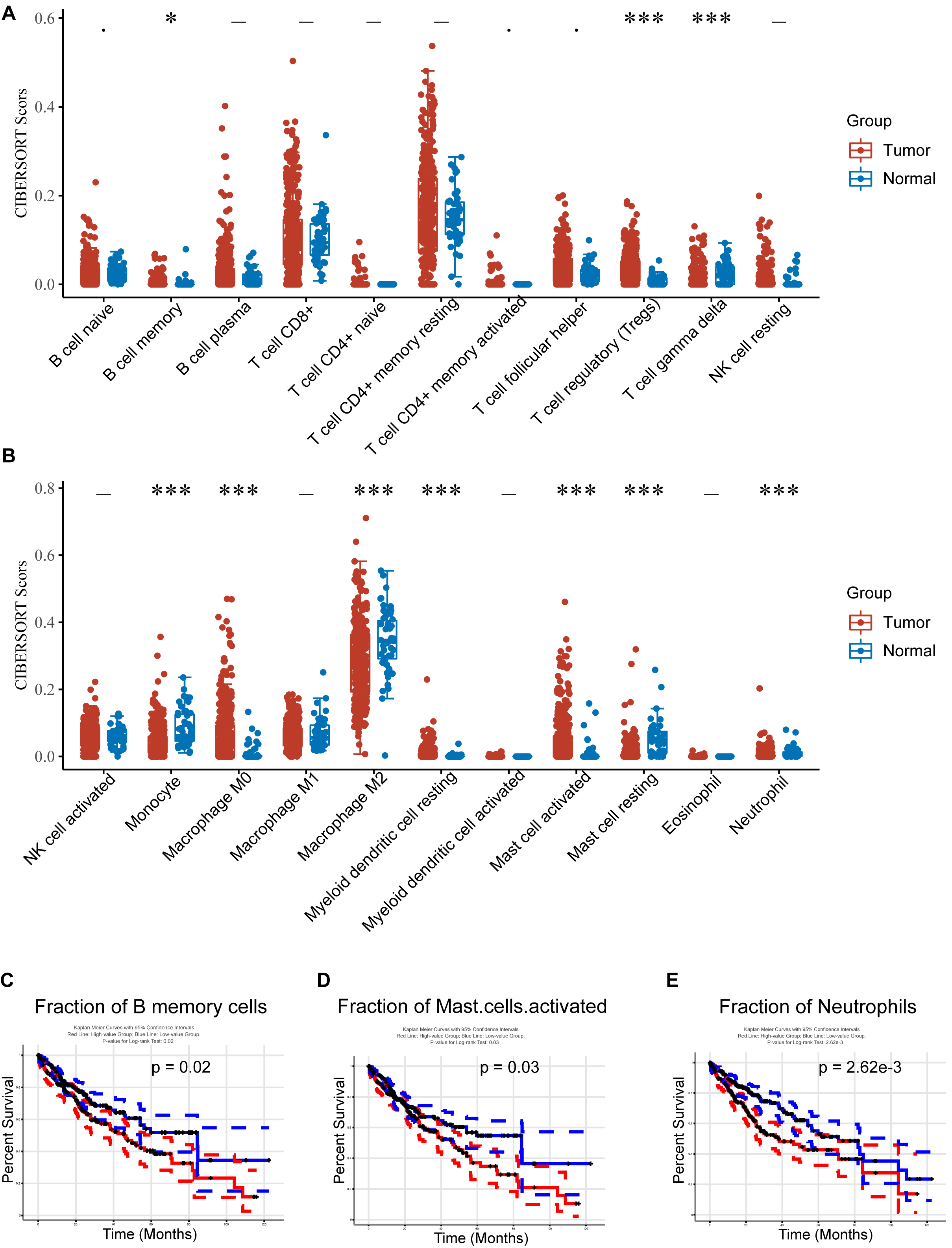

Supplement: Supplementary file 1 [file DataSheet_1.zip › Supplementary Figure2..tif]

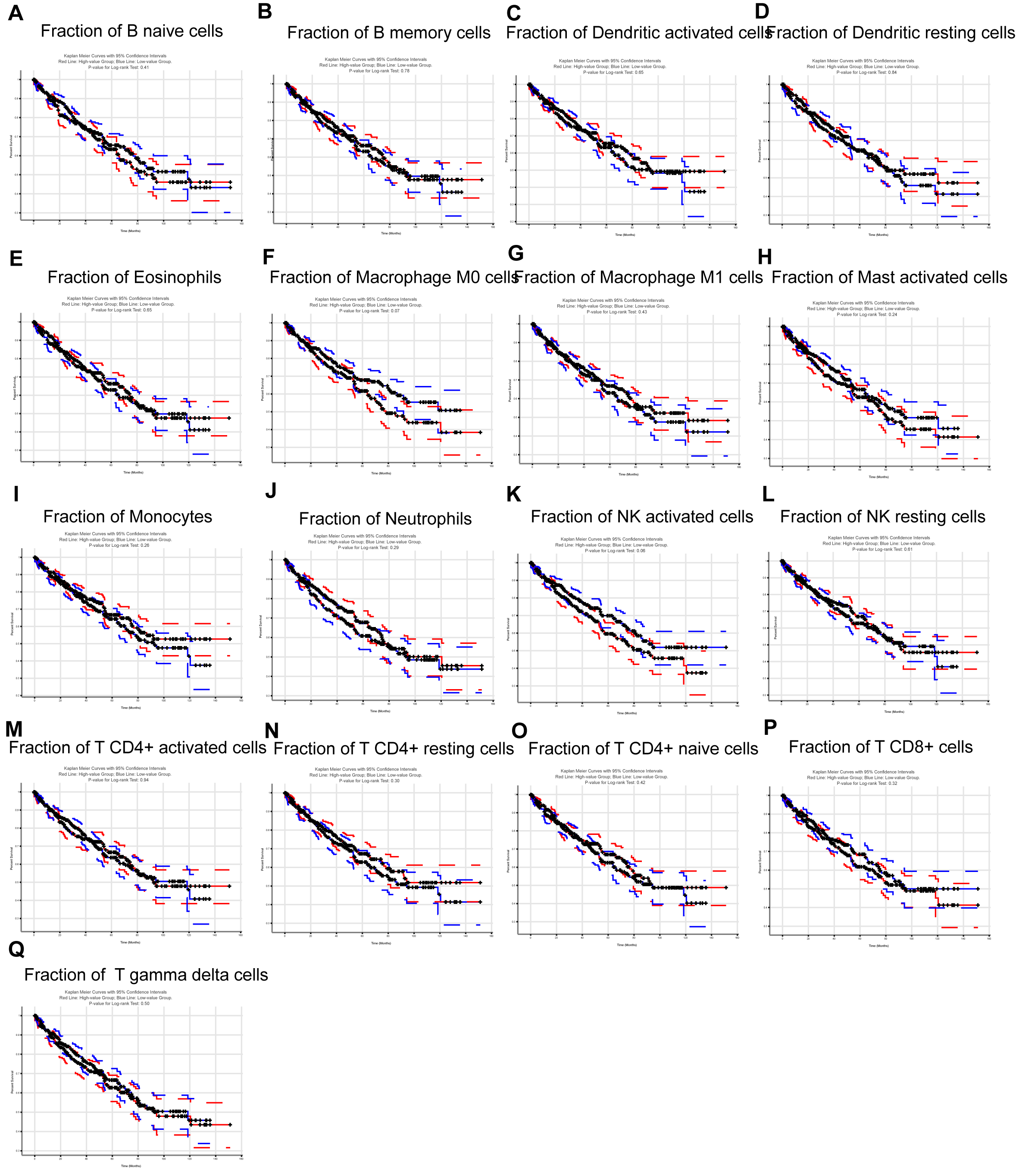

Supplement: Supplementary file 1 [file DataSheet_1.zip › Supplementary Figure3..tif]
